# *tert*-Butyl 3-amino-5-bromo-1*H*-indazole-1-carboxyl­ate

**DOI:** 10.1107/S2414314621006945

**Published:** 2021-07-09

**Authors:** Aravazhi Amalan Thiruvalluvar, Raviraj Kusanur, Makuteswaran Sridharan

**Affiliations:** a Principal (Retired), Kunthavai Naacchiyaar Government Arts College for Women (Autonomous), Thanjavur 613 007, Tamilnadu, India; b Department of Chemistry, RV College of Engineering, Bangalore 560 059, Karnataka, India; University of Aberdeen, Scotland

**Keywords:** crystal structure, 1*H*-indazole, inversion dimer

## Abstract

In the title compound, C_12_H_14_BrN_3_O_2_, the pyrazole and benzene rings are nearly co-planar with a dihedral angle between the rings of 2.36 (5)°. In the crystal, inversion dimers linked by pairwise N—H⋯N hydrogen bonds generate *R*_2_^2^(8) loops. The dimers are linked into a three-dimensional network by weak aromatic π–π stacking inter­actions [centroid–centroid separation = 3.7394 (6) Å] and C—H⋯O and C—H⋯Br hydrogen bonds.

## Abstract 2

In the packing of the title compound, π–π, C—H⋯O, C—H⋯Br, and N—H⋯N inter­actions are present.

## Graphical abstract

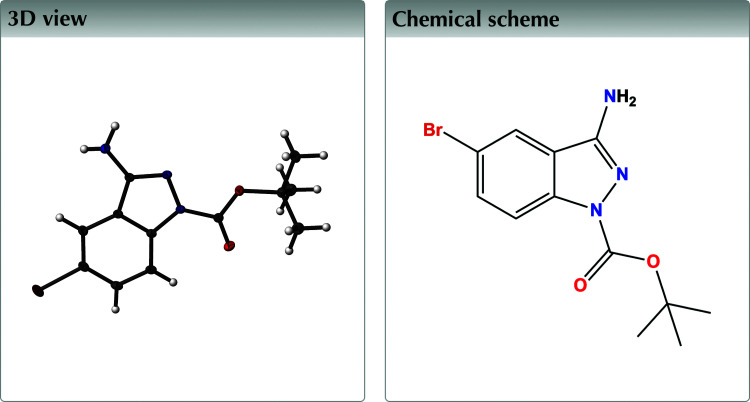

## Structure description

Indazole derivatives possess pharmacological properties against infectious, neurodegenerative and inflammatory disorders and are also good anti-microbial agents (*e.g.*, Kusanur & Mahesh, 2013 ▸). To generate a library of compounds using 3-amino-6-bromo indazole, the boc protection of the ring NH group was carried out to form the title compound. From the crystal data, it is confirmed that, as expected, the boc protection happened only at the ring NH grouping.

**Figure 1:**
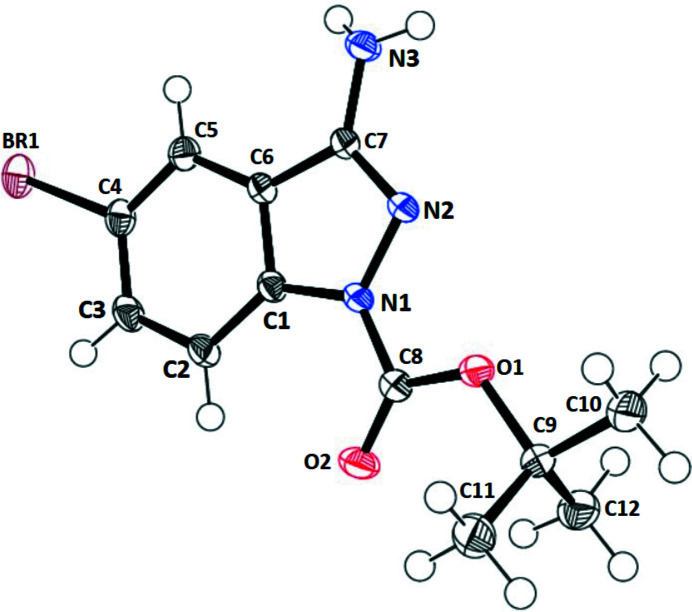
A view of the structure of the title compound with displacement ellipsoids drawn at the 70% probability level.

In this structure (Fig. 1 ▸), the fused pyrazole (N1/N2/C7/C6/C1) and benzene (C1–C6) rings are nearly co-planar, subtending a dihedral angle of 2.36 (5)°. The dihedral angle between the C8/O1/O2 ester group and the fused-ring system is 10.01 (4)°. One of the methyl groups (C10) of the *tert*-butyl substituent lies close to the ester-group plane [displacement = −0.068 (1) Å], whereas C11 and C12 are displaced above and below it. Very weak C2—H2⋯O2, C11—H11*C*⋯O2 and C12—H12*B*⋯O2 intra­molecular inter­actions are present (Table 1 ▸).

**Figure 2:**
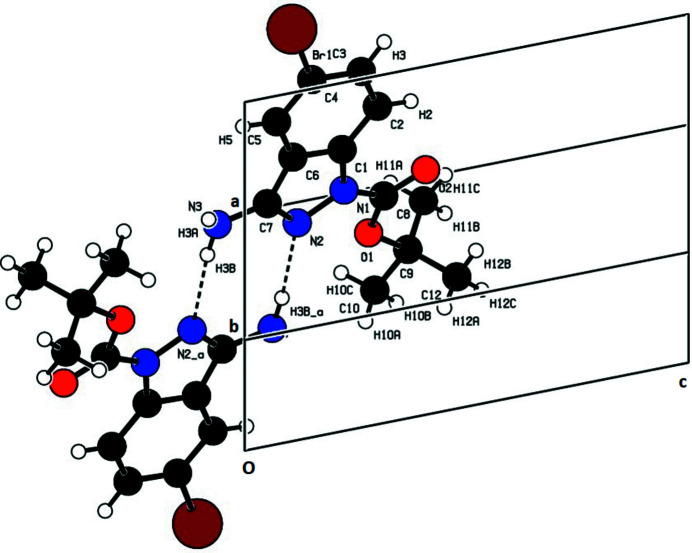
Partial packing viewed along *b*-axis direction showing the 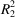 (8) ring motif.

**Figure 3:**
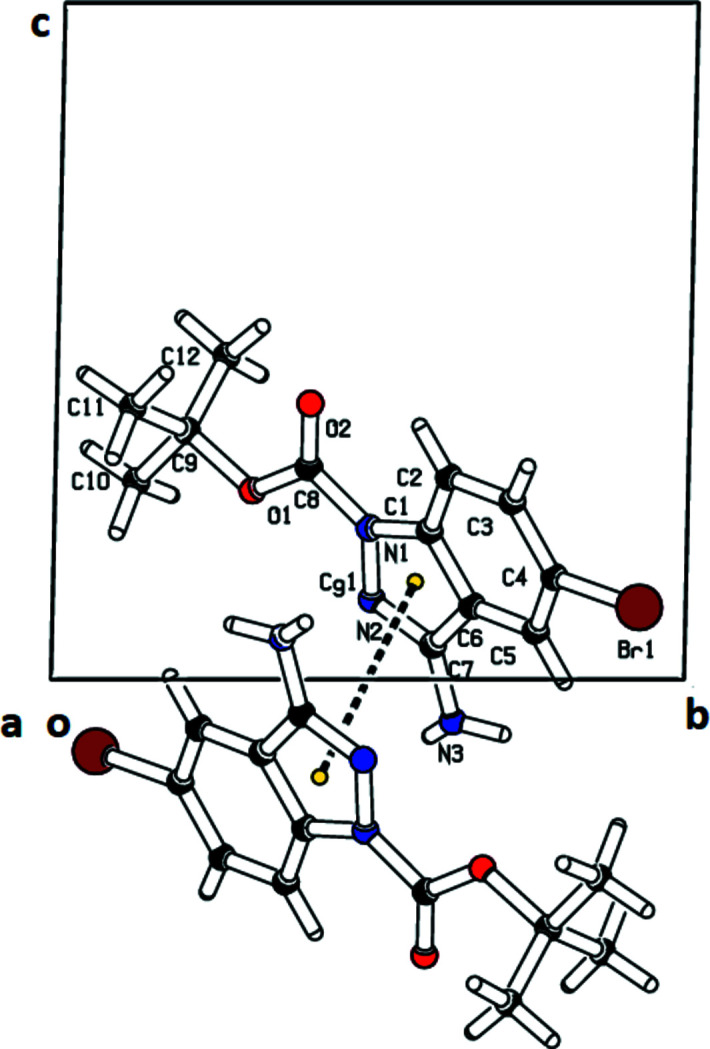
A view of the π–π inter­action along the *a*-axis direction.

In the extended structure, pairwise N3—H3*B*⋯N2 links form centrosymmetric dimers with an 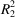 (8) ring motif (Fig. 2 ▸). The dimers are linked into a three-dimensional network by C2—H2⋯O2, C5—H5⋯Br1 and C12—H12*A*⋯O2 hydrogen bonds and a π–π stacking inter­action (Fig. 3 ▸) also occurs with *Cg*1⋯*Cg*1(2 − *x*, 1 − y, −*z*) = 3.7394 (6) Å, where *Cg*1 is the centroid of the pyrazole ring.

## Synthesis and crystallization

**Figure 4:**
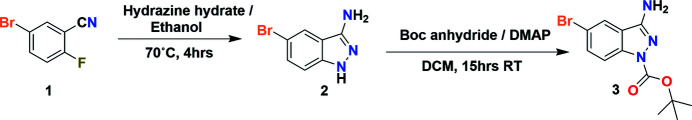
Synthesis scheme for the title compound.

**5-Bromo-1** ***H*** **-indazol-3-amine (1):** To a solution of 5-bromo-2-fluoro benzo­nitrile (1.0 mmol) in ethanol (20 ml) was added hydrazine hydrate (99%) (10.0 mmol). The reaction mixture was heated in sealed tube at 343 K for 4 h and progress of the reaction was monitored by TLC. The reaction mixture was concentrated to dryness. The brown-coloured solid was purified by recrystallization from ethanol solution to afford pale-yellow needles (90%), m.p. 407 K (Fig. 4 ▸).

***tert*** **-Butyl 3-amino-5-bromo-1** ***H*** **-indazole-1-carboxyl­ate (2):** To a solution of compound (**1**) (5.0 mmol) in di­chloro­methane (40 ml) was added DMAP (5.0 mmol). The reaction mixture cooled to 273 K and boc anhydride (5.0 mmol) was added. The reaction mixture was slowly warmed to room temperature and stirred for 15 h. Progress of the reaction was monitored by TLC. The reaction mixture was diluted with di­chloro­methane (50 ml) and washed with water and brine (25 ml), dried over anhydrous sodium sulfate and concentrated. The crude compound was purified by column chromatography (silica gel, 20–30% ethyl acetate in hexa­ne) to afford a gummy solid, which solidifies as transparent crystals after 2 d (62%), m.p. 389 K.

## Refinement

Crystal data, data collection and structure refinement details are summarized in Table 2 ▸.

## Supplementary Material

Crystal structure: contains datablock(s) I, global. DOI: 10.1107/S2414314621006945/hb4388sup1.cif

Structure factors: contains datablock(s) I. DOI: 10.1107/S2414314621006945/hb4388Isup2.hkl

Supporting information file. DOI: 10.1107/S2414314621006945/hb4388Isup3.cdx

Supporting information file. DOI: 10.1107/S2414314621006945/hb4388Isup4.cml

CCDC reference: 2094667

Additional supporting information:  crystallographic information; 3D view; checkCIF report

## Figures and Tables

**Table 1:** Hydrogen-bond geometry (Å, °)

*D*—H⋯*A*	*D*—H	H⋯*A*	*D*⋯*A*	*D*—H⋯*A*
C2—H2⋯O2	0.95	2.46	2.9609 (13)	113
C11—H11*C*⋯O2	0.98	2.38	2.9559 (15)	117
C12—H12*B*⋯O2	0.98	2.46	3.0475 (15)	118
N3—H3*B*⋯N2^i^	0.865 (18)	2.165 (19)	3.0249 (12)	172.8 (16)
C2—H2⋯O2^ii^	0.95	2.62	3.4133 (12)	141
C5—H5⋯Br1^iii^	0.95	3.11	3.8871 (10)	140
C12—H12*A*⋯O2^iv^	0.98	2.62	3.5582 (14)	161

Symmetry codes: (i) 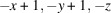 ; (ii) 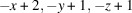 ; (iii) 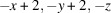 ; (iv) 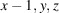 .

**Table 2:** Experimental details

Crystal data	
Chemical formula	C_12_H_14_BrN_3_O_2_
*M*_r_	312.17
Crystal system, space group	Triclinic, *P* 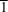
Temperature (K)	100
*a*, *b*, *c* (Å)	5.8281 (2), 10.5313 (3), 11.0917 (3)
α, β, γ (°)	85.954 (1), 78.801 (2), 75.105 (1)
*V* (Å^3^)	645.23 (3)
*Z*	2
Radiation type	Mo *K*α
μ (mm^−1^)	3.18
Crystal size (mm)	0.45 × 0.32 × 0.30

Data collection	
Diffractometer	Bruker SMART APEXII CCD
Absorption correction	Multi-scan (*SADABS*; Bruker, 2012 ▸)
*T*_min_, *T*_max_	0.502, 0.748
No. of measured, independent and observed [*I* > 2σ(*I*)] reflections	19603, 7076, 5872
*R*_int_	0.018
(sin θ/λ)_max_ (Å^−1^)	0.895

Refinement	
*R*[*F* ^2^ > 2σ(*F* ^2^)], *wR*(*F* ^2^), *S*	0.027, 0.070, 1.08
No. of reflections	7076
No. of parameters	174
H-atom treatment	H atoms treated by a mixture of independent and constrained refinement
Δρ_max_, Δρ_min_ (e Å^−3^)	0.59, −0.53

Computer programs: *APEX2* and *SAINT* (Bruker, 2012 ▸), *SHELXS97* (Sheldrick, 2008 ▸), *SHELXL2018/3* (Sheldrick, 2015 ▸), *ORTEP-3 for Windows* (Farrugia, 2012 ▸), *PLATON* (Spek, 2020 ▸) and *publCIF* (Westrip, 2010 ▸).

## full crystallographic data

### Crystal data

**Table:**

C_12_H_14_BrN_3_O_2_	*F*(000) = 316
*M**_r_* = 312.17	*D*_x_ = 1.607 Mg m^−^^3^
Triclinic, *P*1	Melting point: 389 K
*a* = 5.8281 (2) Å	Mo *K*α radiation, λ = 0.71073 Å
*b* = 10.5313 (3) Å	Cell parameters from 2120 reflections
*c* = 11.0917 (3) Å	θ = 2.7–25.5°
α = 85.954 (1)°	µ = 3.18 mm^−^^1^
β = 78.801 (2)°	*T* = 100 K
γ = 75.105 (1)°	Fragment, colourless
*V* = 645.23 (3) Å^3^	0.45 × 0.32 × 0.30 mm
*Z* = 2	

### Data collection

**Table:**

Bruker SMART APEXII CCD diffractometer	5872 reflections with *I* > 2σ(*I*)
φ and ω scans	*R*_int_ = 0.018
Absorption correction: multi-scan (SADABS; Bruker, 2012)	θ_max_ = 39.5°, θ_min_ = 1.9°
*T*_min_ = 0.502, *T*_max_ = 0.748	*h* = −10→10
19603 measured reflections	*k* = −18→18
7076 independent reflections	*l* = −19→19

### Refinement

**Table:**

Refinement on *F*^2^	Primary atom site location: structure-invariant direct methods
Least-squares matrix: full	Secondary atom site location: difference Fourier map
*R*[*F*^2^ > 2σ(*F*^2^)] = 0.027	Hydrogen site location: mixed
*wR*(*F*^2^) = 0.070	H atoms treated by a mixture of independent and constrained refinement
*S* = 1.08	*w* = 1/[σ^2^(*F*_o_^2^) + (0.0327*P*)^2^ + 0.1773*P*] where *P* = (*F*_o_^2^ + 2*F*_c_^2^)/3
7076 reflections	(Δ/σ)_max_ = 0.002
174 parameters	Δρ_max_ = 0.59 e Å^−^^3^
0 restraints	Δρ_min_ = −0.53 e Å^−^^3^

### Special details

**Table:**

Geometry. All esds (except the esd in the dihedral angle between two l.s. planes) are estimated using the full covariance matrix. The cell esds are taken into account individually in the estimation of esds in distances, angles and torsion angles; correlations between esds in cell parameters are only used when they are defined by crystal symmetry. An approximate (isotropic) treatment of cell esds is used for estimating esds involving l.s. planes.
Refinement. The amino –NH_2_ H atoms were located in a difference Fourier map and their positions were freely refined. The C-bound H atoms were placed in calculated positions (C—H = 0.95–0.98 Å) and were refined with *U*_iso_(H) = 1.2*U*_eq_(C) or 1.5*U*_eq_(methyl C).

### Fractional atomic coordinates and isotropic or equivalent isotropic displacement parameters (Å^2^)

**Table:**

	*x*	*y*	*z*	*U*_iso_*/*U*_eq_	
C1	0.90907 (17)	0.59306 (9)	0.21941 (9)	0.01265 (14)	
C2	1.04771 (18)	0.62182 (10)	0.29904 (9)	0.01532 (16)	
H2	1.063744	0.574273	0.374242	0.018*	
C3	1.16027 (19)	0.72288 (10)	0.26285 (10)	0.01661 (17)	
H3	1.255405	0.745757	0.314333	0.020*	
C4	1.13572 (18)	0.79194 (10)	0.15122 (10)	0.01534 (16)	
C5	1.00073 (18)	0.76403 (10)	0.07169 (9)	0.01467 (16)	
H5	0.987132	0.811006	−0.003982	0.018*	
C6	0.88544 (17)	0.66311 (9)	0.10852 (9)	0.01246 (14)	
C7	0.73941 (17)	0.60237 (9)	0.05102 (9)	0.01226 (14)	
C8	0.76837 (18)	0.40037 (10)	0.31328 (9)	0.01421 (15)	
C9	0.61429 (18)	0.20590 (10)	0.36754 (9)	0.01514 (16)	
C10	0.5067 (2)	0.12827 (12)	0.29308 (11)	0.0236 (2)	
H10A	0.356430	0.184090	0.272305	0.035*	
H10B	0.472695	0.051548	0.341495	0.035*	
H10C	0.621570	0.099056	0.217351	0.035*	
C11	0.8457 (2)	0.11997 (11)	0.40268 (11)	0.02108 (19)	
H11A	0.963638	0.090441	0.328004	0.032*	
H11B	0.810398	0.043417	0.450934	0.032*	
H11C	0.912110	0.170719	0.451721	0.032*	
C12	0.4299 (2)	0.26645 (12)	0.47786 (11)	0.0218 (2)	
H12A	0.285095	0.320478	0.450072	0.033*	
H12B	0.498911	0.321461	0.521026	0.033*	
H12C	0.386670	0.196475	0.533695	0.033*	
Br1	1.30179 (2)	0.92671 (2)	0.10637 (2)	0.01982 (3)	
N1	0.78125 (15)	0.49788 (8)	0.22385 (8)	0.01335 (13)	
N2	0.67956 (15)	0.50413 (8)	0.11849 (7)	0.01299 (13)	
N3	0.67793 (17)	0.63640 (9)	−0.06185 (8)	0.01576 (15)	
O1	0.66674 (15)	0.31183 (8)	0.27930 (7)	0.01632 (13)	
O2	0.84393 (16)	0.40048 (8)	0.40767 (7)	0.01973 (15)	
H3A	0.669 (3)	0.7159 (19)	−0.0811 (17)	0.029 (5)*	
H3B	0.575 (3)	0.6006 (17)	−0.0839 (16)	0.025 (4)*	

### Atomic displacement parameters (Å^2^)

**Table:**

	*U*^11^	*U*^22^	*U*^33^	*U*^12^	*U*^13^	*U*^23^
C1	0.0122 (4)	0.0141 (4)	0.0128 (3)	−0.0042 (3)	−0.0033 (3)	−0.0017 (3)
C2	0.0155 (4)	0.0186 (4)	0.0141 (4)	−0.0059 (3)	−0.0051 (3)	−0.0017 (3)
C3	0.0160 (4)	0.0194 (4)	0.0172 (4)	−0.0068 (3)	−0.0054 (3)	−0.0041 (3)
C4	0.0148 (4)	0.0145 (4)	0.0181 (4)	−0.0059 (3)	−0.0020 (3)	−0.0031 (3)
C5	0.0160 (4)	0.0137 (4)	0.0156 (4)	−0.0059 (3)	−0.0031 (3)	−0.0004 (3)
C6	0.0123 (4)	0.0132 (4)	0.0131 (3)	−0.0039 (3)	−0.0038 (3)	−0.0009 (3)
C7	0.0119 (3)	0.0136 (4)	0.0124 (3)	−0.0042 (3)	−0.0032 (3)	−0.0006 (3)
C8	0.0144 (4)	0.0157 (4)	0.0136 (4)	−0.0049 (3)	−0.0041 (3)	0.0008 (3)
C9	0.0158 (4)	0.0163 (4)	0.0147 (4)	−0.0065 (3)	−0.0044 (3)	0.0039 (3)
C10	0.0311 (6)	0.0252 (5)	0.0218 (5)	−0.0178 (4)	−0.0103 (4)	0.0064 (4)
C11	0.0169 (4)	0.0192 (4)	0.0260 (5)	−0.0022 (3)	−0.0055 (4)	0.0031 (4)
C12	0.0161 (4)	0.0275 (5)	0.0193 (4)	−0.0035 (4)	−0.0008 (4)	0.0033 (4)
Br1	0.01951 (5)	0.01624 (5)	0.02640 (6)	−0.00958 (4)	−0.00275 (4)	−0.00328 (4)
N1	0.0151 (3)	0.0157 (3)	0.0120 (3)	−0.0067 (3)	−0.0058 (3)	0.0013 (3)
N2	0.0140 (3)	0.0156 (3)	0.0115 (3)	−0.0055 (3)	−0.0052 (3)	0.0005 (3)
N3	0.0205 (4)	0.0166 (4)	0.0136 (3)	−0.0080 (3)	−0.0077 (3)	0.0022 (3)
O1	0.0216 (3)	0.0177 (3)	0.0137 (3)	−0.0105 (3)	−0.0069 (3)	0.0039 (2)
O2	0.0260 (4)	0.0218 (4)	0.0159 (3)	−0.0096 (3)	−0.0111 (3)	0.0037 (3)

### Geometric parameters (Å, º)

**Table:**

C1—N1	1.3874 (12)	C9—O1	1.4835 (12)
C1—C6	1.4006 (13)	C9—C10	1.5177 (15)
C1—C2	1.4019 (13)	C9—C12	1.5185 (16)
C2—C3	1.3859 (15)	C9—C11	1.5222 (15)
C2—H2	0.9500	C10—H10A	0.9800
C3—C4	1.4039 (15)	C10—H10B	0.9800
C3—H3	0.9500	C10—H10C	0.9800
C4—C5	1.3815 (14)	C11—H11A	0.9800
C4—Br1	1.9021 (10)	C11—H11B	0.9800
C5—C6	1.3954 (13)	C11—H11C	0.9800
C5—H5	0.9500	C12—H12A	0.9800
C6—C7	1.4453 (13)	C12—H12B	0.9800
C7—N2	1.3141 (12)	C12—H12C	0.9800
C7—N3	1.3671 (12)	N1—N2	1.3998 (11)
C8—O2	1.2122 (12)	N3—H3A	0.840 (19)
C8—O1	1.3351 (12)	N3—H3B	0.865 (18)
C8—N1	1.3824 (13)		
			
N1—C1—C6	106.05 (8)	C10—C9—C11	110.23 (10)
N1—C1—C2	132.33 (9)	C12—C9—C11	112.91 (9)
C6—C1—C2	121.59 (9)	C9—C10—H10A	109.5
C3—C2—C1	116.85 (9)	C9—C10—H10B	109.5
C3—C2—H2	121.6	H10A—C10—H10B	109.5
C1—C2—H2	121.6	C9—C10—H10C	109.5
C2—C3—C4	120.95 (9)	H10A—C10—H10C	109.5
C2—C3—H3	119.5	H10B—C10—H10C	109.5
C4—C3—H3	119.5	C9—C11—H11A	109.5
C5—C4—C3	122.74 (9)	C9—C11—H11B	109.5
C5—C4—Br1	118.85 (8)	H11A—C11—H11B	109.5
C3—C4—Br1	118.40 (7)	C9—C11—H11C	109.5
C4—C5—C6	116.38 (9)	H11A—C11—H11C	109.5
C4—C5—H5	121.8	H11B—C11—H11C	109.5
C6—C5—H5	121.8	C9—C12—H12A	109.5
C5—C6—C1	121.49 (8)	C9—C12—H12B	109.5
C5—C6—C7	133.15 (9)	H12A—C12—H12B	109.5
C1—C6—C7	105.28 (8)	C9—C12—H12C	109.5
N2—C7—N3	122.92 (9)	H12A—C12—H12C	109.5
N2—C7—C6	111.54 (8)	H12B—C12—H12C	109.5
N3—C7—C6	125.46 (9)	C8—N1—C1	126.18 (8)
O2—C8—O1	127.43 (9)	C8—N1—N2	122.33 (8)
O2—C8—N1	121.75 (9)	C1—N1—N2	111.27 (8)
O1—C8—N1	110.82 (8)	C7—N2—N1	105.85 (8)
O1—C9—C10	102.25 (8)	C7—N3—H3A	113.2 (13)
O1—C9—C12	109.28 (9)	C7—N3—H3B	118.0 (12)
C10—C9—C12	110.83 (9)	H3A—N3—H3B	117.7 (17)
O1—C9—C11	110.84 (8)	C8—O1—C9	119.35 (8)
			
N1—C1—C2—C3	177.48 (10)	O2—C8—N1—C1	10.52 (16)
C6—C1—C2—C3	0.02 (15)	O1—C8—N1—C1	−169.07 (9)
C1—C2—C3—C4	−0.25 (15)	O2—C8—N1—N2	−175.37 (10)
C2—C3—C4—C5	−0.10 (16)	O1—C8—N1—N2	5.04 (13)
C2—C3—C4—Br1	−178.60 (8)	C6—C1—N1—C8	175.41 (9)
C3—C4—C5—C6	0.68 (15)	C2—C1—N1—C8	−2.34 (17)
Br1—C4—C5—C6	179.17 (7)	C6—C1—N1—N2	0.75 (11)
C4—C5—C6—C1	−0.91 (14)	C2—C1—N1—N2	−177.00 (10)
C4—C5—C6—C7	−177.26 (10)	N3—C7—N2—N1	177.97 (9)
N1—C1—C6—C5	−177.46 (9)	C6—C7—N2—N1	0.84 (11)
C2—C1—C6—C5	0.59 (15)	C8—N1—N2—C7	−175.90 (9)
N1—C1—C6—C7	−0.22 (10)	C1—N1—N2—C7	−1.00 (11)
C2—C1—C6—C7	177.83 (9)	O2—C8—O1—C9	6.31 (16)
C5—C6—C7—N2	176.37 (10)	N1—C8—O1—C9	−174.13 (8)
C1—C6—C7—N2	−0.41 (11)	C10—C9—O1—C8	−179.09 (9)
C5—C6—C7—N3	−0.68 (18)	C12—C9—O1—C8	63.43 (12)
C1—C6—C7—N3	−177.45 (9)	C11—C9—O1—C8	−61.63 (12)

### Hydrogen-bond geometry (Å, º)

**Table:**

*D*—H···*A*	*D*—H	H···*A*	*D*···*A*	*D*—H···*A*
C2—H2···O2	0.95	2.46	2.9609 (13)	113
C11—H11*C*···O2	0.98	2.38	2.9559 (15)	117
C12—H12*B*···O2	0.98	2.46	3.0475 (15)	118
N3—H3*B*···N2^i^	0.865 (18)	2.165 (19)	3.0249 (12)	172.8 (16)
C2—H2···O2^ii^	0.95	2.62	3.4133 (12)	141
C5—H5···Br1^iii^	0.95	3.11	3.8871 (10)	140
C12—H12*A*···O2^iv^	0.98	2.62	3.5582 (14)	161

Symmetry codes: (i) −*x*+1, −*y*+1, −*z*; (ii) −*x*+2, −*y*+1, −*z*+1; (iii) −*x*+2, −*y*+2, −*z*; (iv) *x*−1, *y*, *z*.

## References

[bb1] Bruker (2012). *APEX2*. Bruker AXS Inc., Madison, Wisconsin, USA.

[bb2] Farrugia, L. J. (2012). *J. Appl. Cryst.* **45**, 849–854.

[bb3] Kusanur, R. & Mahesh, R. (2013). *Int. J. Life Pharma. Res.* **3**, 6–10.

[bb4] Sheldrick, G. M. (2008). *Acta Cryst.* A**64**, 112–122. 10.1107/S0108767307043930 18156677

[bb5] Sheldrick, G. M. (2015). *Acta Cryst.* C**71**, 3–8.

[bb6] Spek, A. L. (2020). *Acta Cryst.* E**76**, 1–11. 10.1107/S2056989019016244 PMC6944088 31921444

[bb7] Westrip, S. P. (2010). *J. Appl. Cryst.* **43**, 920–925.

